# Eggshell membrane protein can be absorbed and utilised in the bodies of rats

**DOI:** 10.1186/s13104-019-4306-0

**Published:** 2019-05-09

**Authors:** Ryosuke Matsuoka, Hitoshi Kurihara, Hiroko Yukawa, Ryou Sasahara

**Affiliations:** R&D Division, Kewpie Corporation, Sengawa Kewport, 2-5-7, Sengawa-cho, Chofu-shi, Tokyo 182-0002 Japan

**Keywords:** Eggshell membrane, Protein, Hydrolysate, Net protein utilisation, Rats

## Abstract

**Objective:**

Eggshell membranes, the thin film lining the insides of eggshells, are constituted mostly from protein (eggshell membrane protein, ESM-P). The digestibility and dietary net protein utility of ESM-P are not known. ESM-P functions as a barrier to prevent foreign matter from reaching the egg white and yolk, so it would be expected not to decompose easily by digestion when ingested. We therefore prepared a hydrolysate of the membrane (ESM-H). In this study, we assessed the digestibility and net protein utility of ESM-P and ESM-H in rats.

**Results:**

The digestibility of ESM-P and ESM-H were 87.0% and 94.8%, respectively, significantly lower than that of casein (98.5%). The net protein utility values were 84.7% and 84.6%, respectively, significantly higher than that of casein (75.1%). Digestibility was significantly higher for ESM-H than for ESM-P, but there was no significant difference in net protein utility between ESM-P and ESM-H. These results demonstrated that more than 80% of ESM-P or ESM-H is absorbed and utilised in the bodies of rats.

## Introduction

The eggshells and eggshell membranes of hens’ eggs are little used potential dietary resources. Eggshells are known to be a source of calcium, which can increase bone density [1]. The eggshell membrane, a thin film lining the inside of the eggshell, is constituted mainly of protein. Eggshell membranes have been used as carbon dioxide and heavy metal absorbers [2, 3], and also in sauces such as soy sauce [4]. They have also been reported to have a skin-moisturising effect and to ameliorate knee joint pain [5, 6]. To clarify these and other potential health functions, it is important to elucidate the action mechanisms of eggshell membrane protein (ESM-P). A necessary first step is to establish that the protein is digested, absorbed and utilised in the body. However, the digestibility and net protein utility of dietary ESM-P are not known.

Collagen is another protein reported to have a skin-moisturising effect and to ameliorate knee joint pain. Collagen is known not to be utilised in vivo independently because it contains little tryptophan [7]. It has been speculated that collagen exerts its health functions after absorption following its consumption as part of routine protein intake. In contrast, it is likely that ESM-P will be absorbed and utilised in the body because of its 100% amino acid score.

It has been reported that ESM-P include collagen type I, V, and X, and protein derived from egg white [8, 9]. The net protein utility values of proteins in white have been reported to be higher than those of other protein sources [10].

The aim of this study was to assess the digestibility and net protein utility of ESM-P. Because ESM-P functions as barrier to prevent foreign matter from reaching the egg white and yolk, we thought it likely that the protein would not be easily decomposed by digestion when ingested. We therefore prepared a hydrolysate of eggshell membrane (ESM-H), using enzymatic hydrolysis to convert it to low molecular weight peptides, and assessed its digestibility and net protein utility in addition to those of ESM-P.

## Main text

### Methods

#### Materials

Casein was purchased from Oriental Yeast Co., Ltd. (Tokyo). ESM-P and ESM-H were obtained from Kewpie Corporation (Tokyo). ESM-H was prepared by the alkaline protease digestion of ESM-P. After enzyme inactivation at 90 °C, the hydrolysate was spray-dried ready for testing [11]. The amino acid composition of the samples was measured by Japan Food Research Laboratories (Tokyo; Table 1). Their protein content was calculated by multiplying the nitrogen content determined by the Dumas method [12] by 6.25. The protein content/100 g of casein, ESM-P, and ESM-H were 87.4 g, 99.7 g and 75.4 g, respectively. The mean molecular weight of ESM-H was about 855.

**Table 1:** Amino acid composition of casein, ESM-P or ESM-H (g/100 g)

	Casein	ESM-P	ESM-H
Ile	4.65	3.22	2.46
Leu	8.35	4.54	3.57
Lys	7.23	3.26	2.40
Met	2.47	3.71	2.85
Cys	0.39	9.73	5.95
Phe	4.56	1.54	1.23
Tyr	4.97	1.71	1.47
Thr	3.83	5.50	4.22
Trp	1.10	3.27	2.39
Val	5.83	6.92	5.28
His	2.70	3.95	2.86
Arg	3.33	7.13	5.38
Ala	2.73	2.59	2.21
Asp	6.35	8.19	6.37
Gul	19.7	12.4	9.47
Gly	1.69	5.84	4.50
Pro	9.48	9.36	7.48
Ser	5.09	5.21	4.02

*ESM* egg shell membrane

#### Animals and diets

Eight-week-old male Sprague Dawley rats (245–275 g) were used (n = 6). The animals were kept in metabolic cages (Toyo-Riko Co., Ltd., Tokyo) at 23.1 °C, with 50% ± 2% humidity and a 12-h light (8:00–20:00)/dark cycle.

Test diets were prepared according to the American Institute of Nutrition (AIN)-76 formulation [13]. They included casein, ESM-P, or ESM-H at 10%. The other ingredients were as follows: cornstarch, 15%; cellulose, 5%; mineral mixture (AIN-76), 3.5%; vitamin mixture (AIN-76), 1%; corn oil, 5%; choline bitartrate, 0.2%; and sucrose to 100%. A protein level of 10% was used in this experiment, as described in previous studies [14, 15], because it is easier to assess net protein utility in a low-protein condition, and it allowed the results to be compared with previous data. The animals were pair-fed one of these three diets for 10 days, with ad libitum access to distilled water. We did not check water consumption. But it was reported that positive relation between dietary intake and water consumption [16]. Their faeces and urine were collected for the last 5 days of the test. A fourth group that received no protein was used to calculate the metabolic nitrogen levels for the analysis: the faecal and urinary metabolic nitrogen levels per 5 days for this group were 16.9 ± 0.7 mg and 26.2 ± 1.6 mg/5 day, respectively. The rats were euthanized by CO_2_ gas.

This experiment was conducted in accordance with the Guidelines for Animal Experiments, Law No. 105 and Notification No. 6, of the Government of Japan and the animal experiment rules of the Research and Development Headquarters. This experiment was approved by Ethics committee of Kewpie corporation R&D division (Reference and Permission No. 17-06). The experiment was performed from 5 to 22 December 2017.

#### Analysis

Faecal and urinary protein contents were calculated by measuring the nitrogen (N) content using the Dumas method [12] and multiplying this by the protein conversion factor 6.25. Protein efficiency, digestibility and net protein utility were calculated from the following formulae [17]:

$$ {\text{Digestibility}} = \left[{{\text{ingested N}}-\left({{\text{faecal N}}-{\text{faecal metabolic N}}} \right)} \right]/{\text{ingested N}} \times 100\% $$

$$ {\text{Net protein utility}} = {\text{Digestibility}}-\left( {{\text{ingested N}}-{\text{urinary metabolic N}}} \right)/{\text{ingested N}} \times 100\% $$

#### Statistical analysis

Test results are expressed as mean ± SE. The statistical analyses used Tukey’s test and were performed using SPSS ver. 20 software (Japan IBM Co., Ltd., Tokyo). Differences were considered statistically significant when the *p* value was less than 5%.

### Results

#### Growth parameters

Table 2 summarises the growth parameters. No significant differences were detected in dietary intake among the three groups. The ESM-P and ESM-H groups showed significantly lower weight gain than the casein group. Food efficiency was significantly higher in the casein group than in the ESM-P group, but there was no significant difference between the ESM-P and ESM-H groups.

**Table 2:** Growth variables, faecal and urine nitrogen content in rats fed a diet containing casein, ESM-protein or ESM-hydrolysate

	Casein	ESM-protein	ESM-hydrolysate
Initial body weight (g)	277 ± 3	277 ± 3	277 ± 3
Final body weight (g)	305 ± 2^a^	286 ± 6^b^	296 ± 2^ab^
Body weight gain (g/day)	2.76 ± 0.22^a^	0.868 ± 0.468^b^	1.87 ± 2.4^ab^
Food consumption (g/day)	19.9 ± 0.0	20.0 ± 0.4	20.1 ± 0.7
Food efficiency*	0.136 ± 0.011^a^	0.0421 ± 0.0236^b^	0.0982 ± 0.0177^ab^
Faecal weight (dry g/5 days)	8.57 ± 0.08	9.90 ± 0.73	9.09 ± 0.40
Urine volume (mL/5 days)	62.8 ± 13.0^a^	26.3 ± 8.4^b^	61.1 ± 3.5^a^
Faecal nitrogen (mg/5 days)	40.8 ± 0.9^a^	226 ± 17^b^	101 ± 5^c^
Urine nitrogen (mg/5 days)	397 ± 12^a^	65.0 ± 10.6^b^	187 ± 12^c^

Mean ± standard error (SE) of six rats. Superscript letters indicate a significant difference (Tukey’s test, *p* < 0.05)

*ESM* egg shell membrane

* Food efficiency: body weight gain (g/day)/food consumption (g/day)

#### Faecal and urine nitrogen content

Table 2 summarises the faecal amount, urine volume and faecal and urinary nitrogen content for the three groups. No significant difference in faecal excretion was detected among the groups. Urinary excretion was significantly lower in the ESM-P group than in the casein and ESM-H groups. Faecal and urinary nitrogen content were significantly higher in the ESM-P and ESM-H groups than in the casein group, and significantly higher in the ESM-P group than in the ESM-H group.

#### Digestibility and net protein utility

**Fig. 1:**
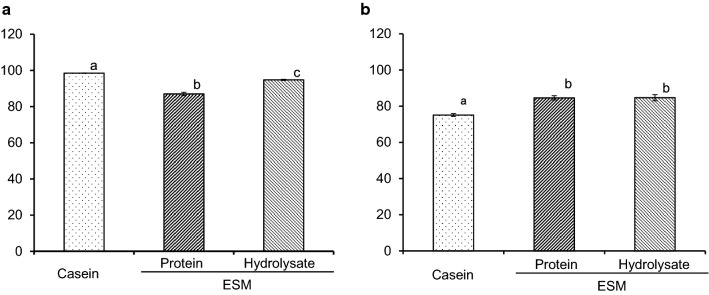
Digestibility (**a**) and net protein utilisation (**b**) of eggshell membrane protein (ESM-P) or hydrolysate (ESM-H) in rats. Mean ± S.E. for six rats. Superscript letters indicate a significant difference (Tukey’s test, *p* < 0.05)

Figure 1 summarises the digestibility and net protein utility. The digestibility of ESM-P and ESM-H were 87.0% and 94.8%, respectively, significantly lower than that of casein (98.5%). The net protein utility values were 84.7% and 84.6%, respectively, significantly higher than that of casein (75.1%). Digestibility was significantly higher for ESM-H than for ESM-P, but there was no significant difference in net protein utility between them.

### Discussion

The results of this study demonstrated that more than 80% of ESM-P is absorbed and utilised in vivo. Follow-up test results showed that the digestibility and net protein utility of ESM-P were 88.8% and 82.4%, and those of ESM-H were 95.1% and 84.7%, respectively (unpublished data). These values were similar to the results of the present study, confirming its reproducibility.

The digestibility and net protein utility values of ESM-P and ESM-H were lower than representative values for white proteins [10]. This may be because of the low content of the essential amino acids phenylalanine + threonine and leucine (Table 1). However, the net protein utility of ESM-P was higher than that of casein, suggesting that the lower absorption of the ESM proteins was due to their physicochemical structures rather than the low content of an essential amino acid. Indeed, their digestibility was significantly lower than that of casein.

Although total nitrogen excretion (faecal + urea) of the casein group was higher than that of the ESM-P and ESM-H groups, the body weight of the casein group was higher than that of the ESM-P and ESM-H groups (Table 2, Fig. 1).

We believe that total nitrogen excretion and NPU affects the body’s protein content. Except for water content, animal bodies consist mainly of protein and fat. It has been reported that egg white protein increases the amount of body protein and decreases the amount of body fat compared with casein in rats [18]. ESM included protein derived from egg white [9]. It was believed that the reason that the body weights of ESM-P and ESM-H groups were lower than that of the casein group was that the body fat in the ESM-P and ESM-H groups was lower than that in the casein group.

ESM-P has been reported to have a skin-moisturising effect and to ameliorate knee joint pain [5, 6]. Similar effects have been reported for hyaluronic acid [19, 20]. Hyaluronic acid is thought to be absorbed after being digested into low molecular weight molecules in the digestive tract by the action of intestinal microbiota [21]. Biokinetic tests have shown it is then delivered to the skin and the knee joints [22, 23].

Hyaluronic acid is a saccharide, but there are proteins that are known to exert similar effects. Collagen has been reported to have similar effects to those of ESM-P. However, collagen can hardly be absorbed independently because of its low content of tryptophan, an essential amino acid. Collagen contains abundant hydroxyproline [7], whereas ESM-P contains collagen [9]. Furthermore, because collagen is a skin and knee joint component, its absorption appears to be an action for supplementing insufficient collagen levels. It is therefore likely that ESM-P exerts one of its effects via a same mechanism. Clarification of this mechanism is likely to require an assessment of ESM-P biokinetics.

In the present study, urine volume was lower in the ESM-P group rats (26.3 ± 8.4 mL/5 days) compared with the volumes for those fed ESM-H and casein. Dietary intake and net protein utilisation were same in the ESM-P and ESM-H groups, but body weight was lower in the ESM-P group than in the ESM-H. Thus, the reason for the lower urine volume in the ESM-P group may have been lower body water content and water intake in this group compared with the ESM-H group. In a previous study, we found a similar urine volume in rats fed milk whey (27.3 ± 4.3 mL/5 days) [10]. In that study, the nitrogen content in the urine was 275 ± 26 mg/day [10]; in the present study, the urine nitrogen content in the ESM-H group was 187 ± 12 mg/day, suggesting that there was no problem in the nitrogen excretion capacity in the rats fed ESM-P.

There are subjects in utilisation of ESM-P in foods, etc. expecting its functions for health. For use in foods, it may be necessary to digest eggshell membrane into low molecular weight molecules for efficient absorption, given its lower digestibility than casein [5], and to improve its flavour and/or physical properties. It may be possible to utilise the protein in supplements, although this would depend on its effective dose.

### Conclusions

In this study, ESM-H showed higher digestibility than ESM-P, but their net protein utility was nearly the same. It is therefore likely that their net protein utility is influenced by their amino acid composition rather than their digestibility. The potential use of ESM-P and ESM-H in food and health products has been little studied. The results of this study should contribute to consideration of ESM-P and ESM-H as a new functional food ingredient.

## Limitations

Clarification of this mechanism is likely to require an assessment of ESM-P biokinetics. It would be necessary first to assess the absorbability and receptor-binding capacity of ESM-P in the small intestine, for example, to establish its action in the digestive tract.

## Data Availability

All data generated or analysed during this study are included in this published article.

## Abbreviations

ESM-P: eggshell membrane protein
ESM-H: eggshell membrane hydrolysate
AIN: American Institute of Nutrition
